# Case series demonstrating in vivo MR safety of stainless steel (Chinese/Ring) IUDs

**DOI:** 10.1259/bjrcr.20210165

**Published:** 2022-01-07

**Authors:** Shailin Thomas, Nicole Hindman

**Affiliations:** 1 Department of Radiology, NYU School of Medicine, New York, USA

## Abstract

Intrauterine devices (IUDs) are one of the most common forms of long-term contraception used by patients around the world. Many studies have been performed over the past few decades demonstrating the safety of many common hormonal and metallic intrauterine devices in Magnetic Resonance (MR) imaging; however, the stainless steel ring IUD (often termed the “Chinese” IUD) is still considered MR Unsafe. This device was used in the 1980s and 1990s in China, where as many as 60 million women in China were using an IUD by 1988, and approximately 90% of those were stainless steel ring IUDs. In a major metropolitan area hospital such as ours with a large immigrant population, we encounter females with this ring IUD several times a year. As this population ages, the need for medical care (and concomitantly, MR imaging) is projected to increase. The purpose of this case review is to examine the imaging and clinical course of patients with stainless-steel ring intrauterine devices who safely received 1.5T Brain MR scans at our institution for clinically necessary diagnostic imaging.

## Introduction

Intrauterine devices (IUDs) are one of the most common forms of long-term contraception used by patients around the world. Usage is estimated at approximately 8% in Europe and North America and in Eastern and South-Eastern Asia, usage is 18.6% and IUDs are the most common contraceptive used.^1^ Since their introduction in the mid-Twentieth Century, IUDs have enabled many patients to safely, and effectively, prevent pregnancy. The long-acting effect and reversibility of intrauterine devices make them a good option for females of reproductive age wishing to temporarily delay pregnancy. Because of their widespread use, IUDs are commonly encountered in the MR environment, where the predominant clinical MR safety concern is regarding heating of the implant and theoretical displacement from the uterus (rendering the contraceptive effect nil).^2^

There are four major international organizations that evaluate standards for MR performance and safety including the National Electrical Manufacturer’s Association (NEMA), the International Electrotechnical Commission (IEC), The International Commission on Non-Ionizing Radiation Protection (ICNIRP) and the American Society for Testing and Materials (ASTM International). Of these, the ASTM International standardizes medical device testing, labeling and terminology (including, but not limited to F2503, F2052 and F2189). Guidelines for MR safety practices have been issued by several radiologic societies, including the American College of Radiology (ACR), Canadian Association of Radiologists and European Union.^3^ Despite these guidance documents, situations arise outside of those defined by Instructions For Use (IFUs), whereby patient care needs necessitate re-evaluation of prior MR safety concerns.^3^ These exceptional situations arise in cases where a medical device is used differently to how the manufacturer has instructed (termed “Off-label” usage by agencies such as the Federal Drug Administration (FDA) and Medicines and Healthcare products Regulatory Agency (MHRA)). These “off-label” cases occur in instances where the MR Safety Officer/radiologist/physicist in conjunction with the referring physician use scientific rationale and sound medical evidence and appropriate consent to tailor care to the individual patient.

The majority of intrauterine devices are made entirely of plastic and release hormones to prevent pregnancy. These hormone-based plastic IUDs are considered MR Safe.^3^ However, some have discrete metallic components (such as ParaGard, which contains copper) that help induce the contraceptive effect. Even a slightly ferromagnetic implantable device can move or heat when introduced to a magnetic field, which can both interfere with the proper functioning of the device and theoretically pose a safety hazard for the patient.^4–6^ However, studies have been performed over the past few decades demonstrating the safety of many common hormonal and metallic intrauterine devices in MR imaging.^2,3,7,8^ The conditional safety of many other commonly encountered metallic implants (*e.g.,* bullets embedded in vertebral bodies, cardiac and coronary stents, temporary epicardial pacing wires etc.) has been shown *in vivo* when scanned under controlled situations (*i.e.,* utilizing whole body SAR less than 2 W/kg with a maximum of 15 min per sequence).^3^ Theoretically, these commonly encountered metallic implants were thought to be unsafe in laboratory *ex vivo* tests (due to the mild heating or displacement observed when scanned out of the body) but this theoretical lack of safety was subsequently disproved in observational *in vivo* studies.^3^

One intrauterine device in particular has been deemed MR unsafe according to current imaging consensus statements.^3,9^ This device, sometimes referred to as the “Chinese ring IUD,” is a stainless-steel ring used in China during the 1980s and 1990s. In part prompted by the country’s former one-child policy, as many as 60 million women in China were using an intrauterine device by 1988, and approximately 90% of those were stainless-steel rings.^10^ While these devices are no longer used in new patients, many females still have them, likely because they were designed without a removal string, making them difficult to extract.^11^ The classification of the stainless-steel ring device as MR unsafe is based on a recent study performed by Bussman et al.^9^ The *ex vivo* study found that both displacement force and torque were significantly higher for the stainless-steel devices when introduced to a magnetic field. The stainless-steel devices exhibited a displacement force of 7.6N and a high degree of torque, while all other metallic IUDs tested exhibited a displacement force of 0.5 mN and no torque effect.^9^ The study also found the stainless-steel ring produced an artefact many times larger than other metallic IUDs, with the artefact on gradient echo up to 200 mm in diameter and on spin echo up to 150 mm in diameter.^9^ These findings (showing a theoretical translational force of 7.6N on the ring IUD) are concerning in theory, because it has been shown that a conventional (non-ring) IUD can perforate the uterus at forces from 20 to 54N, and it is unclear how that extrapolates to a ring IUD.^12^ For reference, typical insertion forces for a conventional (non-ring) IUD range from 1.5 to 6.5N, and removal forces range from 1 to 5.8N.^12^ In clinical practice, however, it is shown extensively that the shape and design of these ring IUDs require significantly greater removal force, such that even in experienced hands, one-third of these devices need to removed in the operating room, as opposed to in the office setting.^13^ Therefore, it is unlikely that these ring IUDs will be displaced from the uterus during an MRI procedure.

While the Bussmann study demonstrates that *ex vivo* MR imaging of stainless-steel ring intrauterine devices move in the magnetic field and generate an artefact greater than other IUD types, it is unclear that these differences are clinically significant. As an important corollary, Maralani et al 2019 describe the evolution of MR Safety as regards pacemakers and defibrillators (nonconditional or legacy cardiac implantable electronic devices (CIEDs)). These CIEDs were previously classified “MR Unsafe” and now are often able to be scanned under specific circumstances (including using a MRI field strength≤1.5T, ensuring a lack of broken leads and having a certified ACLS provider available to monitor and evaluate the CIED pre- and post MRI).^3^ Similarly, for the Chinese ring IUD, refusal to perform an MRI exam for a theoretical concern of displacement or heating of the ring IUD based only on *ex vivo* data can have important adverse clinical consequences. In a major metropolitan area hospital such as ours with a large immigrant population, we encounter females with this ring IUD several times a year. As this population ages, the need for medical care (and concomitantly, MR imaging) is projected to increase. The purpose of this case review is to examine the imaging and clinical course of patients with stainless-steel ring intrauterine devices who safely received 1.5T Brain MR studies at our institution for clinically necessary diagnostic imaging.

## Methods

This study was IRB approved as a Quality Improvement study, with waiver of informed consent. Our hospital database was retrospectively searched from 1 January 2014 to 1 April 2021 for radiology MRI reports that included either “circular IUD” “ring IUD” or “Chinese IUD” in the body or impression. Clinical indication for each scan, the type of MR performed, the scan time, and the radiologist’s impression were all recorded. Complications during and after the scans, as well as device movement, were assessed via technology notes and follow-up medical records, including imaging and clinical notes.

## Results

**Figure 1.**
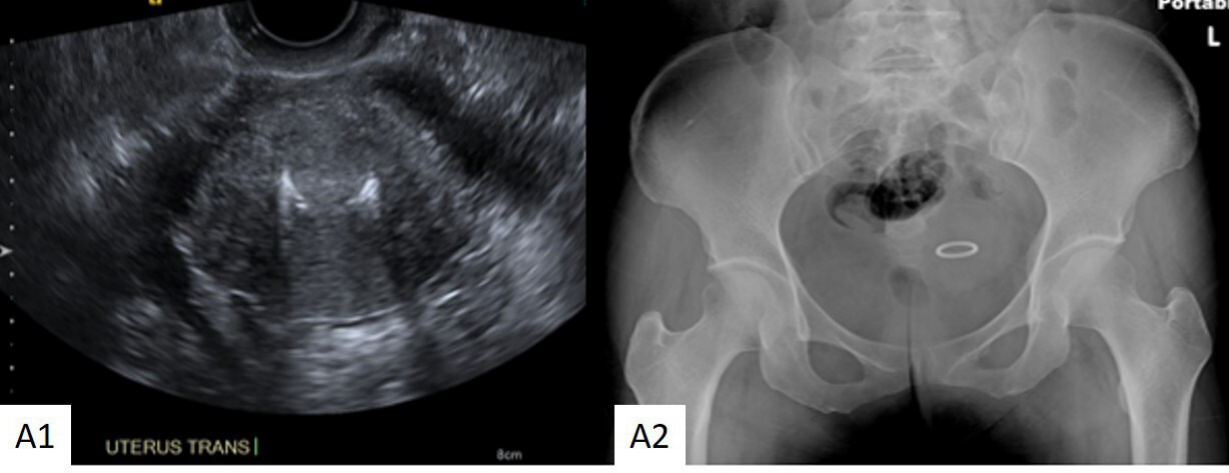
Appearance of Ring IUD 48-year-old female with a ring IUD. A1: Transverse ultrasound image through the uterus demonstrates a ring IUD within the endometrial cavity. A2: Pelvic AP image demonstrates the appearance of the ring IUD in the same patient.

**Figure 2.**
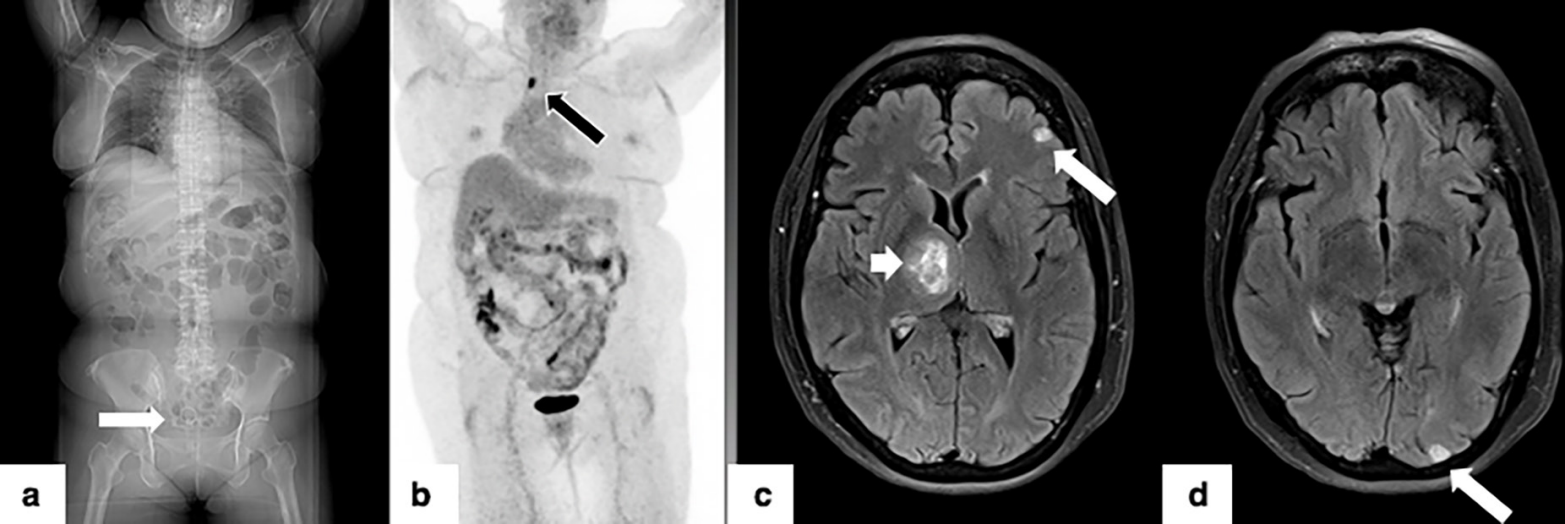
63F with non-small cell lung cancer status post right middle lobectomy and systemic therapy 3 years prior to presentation. Scout images (2a) from PET-CT demonstrate a ring IUD (white arrow) within the pelvis. PET images (2b, black arrow) demonstrate recurrence in the right hilum. Axial post-contrast *T_1_* weighted images of the brain demonstrate multiple metastatic lesions (2c and 2d white arrows) with a dominant mass in the right centrum semiovale and thalamus (2c, small white arrow). The MRI was necessary for pre-operative planning for γ knife therapy.

Of the 56 patients, all Chinese female immigrants, who underwent various radiographic imaging studies noting the presence of a ring intrauterine device, eight patients (aged 47–84 years, average age 58.8) underwent 1.5T MRI (Siemens Aera, Siemens Avanto or GE Optima). The MRIs were obtained after informed consent and risk-benefit discussion between the MR Safety Officer with the referring physician, noting the urgent need for an MRI and the inability to answer the clinical question with other modalities (such as a CT or ultrasound). All eight MRIs occurred without adverse event. The typical appearance of the ring (Chinese) IUD is shown in Figure 1. Each of the eight patients underwent a Brain MRI, of which 2 of 8 were done with contrast to evaluate for metastatic disease in the brain (Figure 2), and 1 of 8 had two sequential studies (a Brain MRI without contrast and a Neck MRI with and without contrast) to evaluate for carotid artery dissection and stroke, after a CT angiogram was unable to answer this clinical question. Another study was performed to evaluate for lymphoma or stroke in a patient with retinal detachment. Clinical indications for the MR imaging studies are noted in Table 1. Scan time for the brain MRIs averaged 20–30 min, with the combined Brain and Neck MRI taking 30 min. None of the patients complained of heating or discomfort during the MR examination, and none of the patients stopped the MR scan for any reason during the study. Of the eight patients, five had follow-up pelvic ultrasounds or pelvic radiographs demonstrating a stable appearance of the devices in the uterus; three had no additional imaging of the pelvis.

**Table 1.** 

	Type of Study	Indication	Impression
Patient 1, age 63	Brain MR with contrast	Evaluate brain metastases, primary lung carcinoma	Interval development of diffuse metastatic disease
Patient 2, age 59	Brain MR with contrast	Evaluate brain metastases	No evidence metastatic disease
Patient 3, age 63	Brain MR w/o contrast	r/o stoke	Findings worrisome for acute infarction
Patient 4, age 84	Brain MR w/o contrast	Dementia, evaluate chronic sub dural hemorrhage	Volume loss/age related atrophy, no sub dural hemorrhage
Patient 5, age 47	Brain MR w/o contrast	Uveitis and retinal detachment, concern for lymphoma/stroke	No evidence for acute stroke or lymphoma
Patient 6, age 59	Brain MR w/o contrast	Trauma, CT worrisome for SAH	No evidence for SAH, chronic age-related degeneration
Patient 7, age 47	Brain MR w/o contrast	r/o stroke	Microvascular disease
Patient 8, age 47	Brain w/o and Neck MR w and w/o contrast	Dystonia/partial seizure, concern for carotid artery dissection on CTA	No carotid artery dissection; parieto-temporal volume loss and cortical scarring

## Discussion

The findings of this case review suggest that stainless-steel ring intrauterine devices may be safer for MR imaging than previous theoretical physics research has posited. All eight patients with stainless-steel intrauterine devices who underwent 1.5T Brain MRIs did so without incident, and all follow-up evaluations demonstrated that the devices were not displaced from the uterus during the scans. The scans provided critical, clinically relevant information for these patients, influencing both diagnosis and treatment course. 2/8 patients had important positive findings seen on MRI that were not appreciated on CT, including evidence for acute infarction and metastatic disease. The other 6/8 patients had important negative findings, such as the absence of a subdural hemorrhage or absence of a subarachnoid hemorrhage (the concern for which was preventing administration of necessary anticoagulation therapy for other medical problems). Other papers have suggested removing the ring IUD as a preemptive way to obtain the MR study; however, this is not a realistically feasible option in a critically ill patient, particularly as these circular IUDs are difficult to remove.

While the study by Bussman et al has served as an underlying justification for declaring these devices MR Unsafe,^9^ the cases evaluated here suggest that an MR Unsafe classification is unwarranted when imaging the brain with 1.5T MRI. The displacement force and torque on the stainless-steel devices were insufficient to move the devices out of the uterus or to cause the patients any discomfort during or after the scans. The most likely explanation for this discrepancy is that the displacement force and torque placed on a stainless-steel device in a patient is not substantial enough to dislodge the device from its place embedded in the endometrium of the uterus when imaging the brain at 1.5T. It is important to note, however, that the force and torque experienced by the device is highly dependent on both the system used and location of the device in the system. For example, in the Bussmann study at the location of *ex vivo* measurement on the 3T system used, the B0 field (roughly proportional to the square root of translational force) was 1.8T and the field gradient was 4.5 T/m, such that the product of field strength and field gradient (roughly propotional to torque) was 8.1 T^2^/m, while in our study we can expect that at the device location (approximately 0.6m from isocenter) B0 should be approximately 1T and field gradient should be approximately 2 T/m for a field-gradient product of approximately 2 T^2^/m.

Stainless-steel intrauterine devices are becoming increasingly uncommon, as they are no longer used in new patients. However, there are patients who still have these devices from the 1980s and 1990s when they were commonplace in some countries, and many of these patients may have clinical indications for MR imaging at some point in the future. Many of these females are now in their late 40s and early 70s, which indicates that radiology practices will continue to see patients with stainless-steel ring IUD for years to come that require MR imaging. Our experience with these patients suggests that the determination of these devices as MR unsafe should be revisited, and that practices that see such patients should consider performing 1.5T MR scans if the scans are clinically necessary and no imaging alternative will suffice.

While more systematic study of the safety of stainless-steel ring intrauterine devices is difficult due to the relative paucity of patients with such devices, further study of the clinical safety and utility of MR imaging in patients with these devices is warranted. In particular, clinical evaluation of stainless-steel device safety in MR imaging at 3T *in vivo* could further elucidate the types of MR scans these patients can receive, and further investigation of pelvic MR imaging with these devices could clarify the extent to which the additional artefact impacts the clinical utility of such studies.

## Learning points

- Many studies have shown that common hormonal and metallic intrauterine devices are safe to scan with MR imaging; however, the stainless-steel ring IUD (often termed the “Chinese” IUD) is still considered MR unsafe.
- These stainless-steel ring IUDs were placed in the 1980s and 1990s, and the females who received these ring IUDs are now in their late 40s to 80s, and their need for medical care and medical (MR) imaging is increasing.
- Removing the ring IUD is technically difficult and often not feasible prior to obtaining a clinically necessary MR study.
- In this retrospective study, eight patients with ring IUDs safely underwent clinically necessary Brain MR imaging studies at 1.5T without adverse event.
- The information from these clinically important MR imaging studies allowed pre-operative planning for Gamma-knife brain surgery in one patient and avoided an unnecessary risky interventional neuroradiology study in another.
- These results suggest that the determination of ring IUDs as MR unsafe should be revisited and that the practices see such patients should consider performing 1.5T MR scans if the scans are clinically necessary and no imaging alternative will suffice.
